# Use of Taxonomic and Trait-Based Approaches to Evaluate the Effect of Bt maize Expressing Cry1Ie Protein on Non-Target Collembola: A Case Study in Northeast China

**DOI:** 10.3390/insects12020088

**Published:** 2021-01-21

**Authors:** Bai-Feng Wang, Feng-Ci Wu, Jun-Qi Yin, Zhi-Lei Jiang, Xin-Yuan Song, Gadi V. P. Reddy

**Affiliations:** 1 Jilin Provincial Key Laboratory of Agricultural Biotechnology, Agro-Biotechnology Research Institute, Jilin Academy of Agriculture Sciences, Changchun 130033, China; wbfwangbaifeng@163.com (B.-F.W.); fengyue36@163.com (F.-C.W.); yin_junqi@163.com (J.-Q.Y.); jiang1891@aliyun.com (Z.-L.J.); 2 USDA-ARS-Southern Insect Management Research Unit,141 Experiment Station Rd., P.O. Box, 346 Stoneville, MS 38776, USA; gadi.reddy@usda.gov

**Keywords:** transgenic *cry1Ie* maize, collembolan diversity, morphological trait, redundancy analysis

## Abstract

**Simple Summary:**

Bt crops have been planted globally since the first commercial Bt maize was cultivated in the United States in 1996. Bt protein from Bt crops can be released to the soil and may potentially affect the non-target soil fauna. Collembola are one of the three most ubiquitous and abundant soil fauna, they have been widely used as indicators of environmental pollution, IE09S034 is a new *cry1Ie* maize breed independently developed by China, and Northeast China is the most important location for maize production in China. Therefore, this study aimed to clarify whether non-target soil Collembola were influenced by the cultivation of *cry1Ie* maize in Northeast China. Our results showed that maize variety had no significant effects on collembolan abundance, diversity, and morphological trait, indicating that two years cultivation of *cry1Ie* maize does not have a bad influence on Collembola in Northeast China.

**Abstract:**

To evaluate the effect of Bt maize expressing Cry1Ie protein on non-target soil Collembola, a two-year field study was conducted in Northeast China. Bt maize line IE09S034 and its near isoline Zong 31 were selected as experimental crops; we investigated the collembolan community using both taxonomic and trait-based approaches, and elucidated the relationship between environmental variables and the collembolan community using redundancy analysis (RDA).The ANOVA results showed that maize variety neither had significant effect on the parameters based on taxonomic approach (abundance, species richness, Shannon–Wiener index, Pielou’s evenness index), nor on the parameters based on trait-based approach (ocelli number, body length, pigmentation level, and furcula development) in either year. The results of RDA also showed that maize variety did not affect collembolan community significantly. These results suggest that two years cultivation of *cry1Ie* maize does not affect collembolan community in Northeast China.

## 1. Introduction

Bt crops have been planted globally since the first commercial Bt maize was cultivated in the United States in 1996 [1]. Bt protein from these crops is released to the soil through several ways, such as root exudates [2,3,4], pollen [5,6], and litter [7,8,9], potentially affecting the soil fauna [10,11,12,13]. Furthermore, the properties of the Bt crops may have changed owing to the transformation of exogenous genes, for example, Saxena and Stotzky found Bt corn had a higher lignin content than non-Bt corn [14], Fang et al. reported that the content of lignin and total organic carbon in Bt maize were higher than that in non-Bt maize [15]. So, the fungal and bacterial populations in microcosms could be influenced by the lignin and nitrogen content in composting conditions [16,17,18], which may ultimately influence the non-target soil fauna [4].

Collembola are one of the three most ubiquitous and abundant members of the soil fauna, having densities of 10^4^–10^5^ per m^2^ in most terrestrial ecosystems [19], and may even reach several million individuals per m^2^ [20]; the number of species is also amazing, with nearly 9000 species having been reported worldwide [21]; and they are sensitive to environmental changes [22,23], so Collembola have been widely used as indicators in evaluating environmental pollution in soil [24,25,26]. Furthermore, different functional groups of Collembola live in different soil layers, usually with different feeding habits and moving patterns, and have evolved unique morphological traits. For example, the euedaphic group (typically represented by the families Onychiuridae and Tullbergiidae)are permanent soil-dwellers, with poor mobility, that utilize fungi from plant root as their food source, are usually white in color, have no ocellus, and the antennae and furculae are underdeveloped; in contrast, the semi-edaphic group (typically represented by the family Isotomidae) that live in superficial soil layer and leaf litter, with normal mobility, utilize fungi from fragmented leaf litter as their food sources, are usually colored but without a pattern, have fewer ocelli, and the antennae, furculae and other appendages are moderately developed; epedaphic group (typically represented by the families Entomobryidae and Sminthurididae) that live at the surface and on vegetation, with strong mobility, and typically utilize fungi from fresh leaf litter as their food source, are usually colored with patterns, have relatively more ocelli, and more highly developed appendages [27]. The changes in these morphological traits are useful in assessing effects of environmental change on the collembolan community [25,28,29,30,31]. So, such a trait-based evaluation approach could complement the taxonomic approach for assessing the impacts of transgenic crops on collembolan community structure [32]. Since the cultivation of Bt crops is increasing, it is important to study its effects on soil Collembola using the combination of taxonomic and trait-based approaches at the species level.

IE09S034 is a new *cry1Ie* maize breed independently developed by the Institute of Plant Protection at the Chinese Academy of Agricultural Sciences [33]; it has a high resistance to Asian maize borer and cotton bollworm [34]. Studies have examined the effects of transgenic *cry1Ie* maize on non-lepidopteran pests and arthropod natural enemies on the above-ground parts of the crop, and have found no impacts [35,36]. Our previous paper also did not show any impacts of *cry1Ie* maize cultivation on soil fauna community when Collembola were analyzed as one group [37]. However, Collembola are extremely abundant, with 17 species reported in the black soil region of Northeast China [32], and this region is the most important location for maize production in China, with IE09S034 as a potential variety to be planted in this region in the future. Therefore, it is of great significance to study the effects of *cry1Ie* maize IE09S034 cultivation on soil collembolan composition and diversity at the species level to clarify the effect and its mechanism more precisely in this region.

In this study, *cry1Ie* maize line IE09S034 and its near isoline Zong 31(without Bt protein) were selected as the experimental crops. We investigated the collembolan community and the environmental variables in the crop field where the two maize varieties were grown. The effects of maize variety on collembolan abundance and some diversity parameters were analyzed using taxonomic approach, on some collembolan morphological trait attribute values were analyzed using trait-based approach. The relationship between environmental variables (including the cultivation of IE09S034) and the collembolan community were elucidated using redundancy analysis. Our findings help clarify whether non-target soil Collembola are influenced by the cultivation of *cry1Ie* maize.

## 2. Materials and Methods

### 2.1. Source of Maize Varieties

Bt maize IE09S034(Bt) and its corresponding non-transformed maize variety Zong 31 (CK) were selected as experimental material in this study. All seeds used in the experiment were provided by the Institute of Crop Sciences.

### 2.2. Experimental Design

The study area was chosen in experimental fields of the Jilin Academy of Agricultural Sciences in Gongzhuling City, China (43°19’ N, 124°29’ E). Conventional maize variety Zhengdan 958 was generally cultivated in the study field for three years prior to 2014.

Maize seeds were manually sown on 7 May 2014, and 6 May 2015, kept apart at distance of 20 cm between the two adjacent maize plants in the same ridge, and 50 cm between the two adjacent ridges. There were six maize plots in two rows, in which three were planted with Bt maize, and another three were planted with Zong 31 maize. The same maize varieties were not planted in the adjacent plots, and they were planted in the same plot in 2014 and 2015. Plot dimensions were 10 m wide and 15 m long, different plots were separated by a 2-m-wide bare strip. The maize plants were harvested when matured on 30 October in both years.

### 2.3. Sample Collection

Collembola were sampled at six maize growth stages during the two-year experiment: “before sowing” stage, “3rd leaf” stage, “elongation” stage, “silking” stage, “physiological maturity” stage, and “harvesting” stage. As we previously described [36], at each sampling time we selected five sampling points within each plot from which to extract soil fauna. Five new sampling points were randomly selected for each sampling time in each plot.

At each point, we collected Collembola from around the maize roots using a soil auger (15 cm in diameter, 10 cm in height). About 200 mL of soil at each point was collected into a plastic bag. Simultaneously, the maize plants near the sampling point were dug out, and the roots were cut off. The soil and the roots were taken back to laboratory for analysis.

The 200-mL soil sample was divided into three parts; 190 mL was placed on a size 20-mesh screen over a Macfadyen extractor funnel [38], and the soil invertebrates were extracted for seven days at room temperature (about 25 °C). The soil Collembola moved down through the funnel and dropped into a collection bottle below with 95% alcohol. The number and species of the Collembola were counted under a microscope (Olympus SZ 51, Japan) at 40 X magnification. Collembola were identified to morphological species according to previously published papers [39,40,41,42]. Additionally, 5 mL of the soil was dried in an oven at 105 °C, and the soil water content was measured, while 5 mL was mixed with an equal volume of 0.01 mol/L CaCl_2_, shaken for 5 min, and held under constant conditions for 24 h, and then the soil pH value was measured with a pH meter (Dynamica, UK). The root was washed clean, dried in an oven at 55 °C, and the root biomass was measured.

### 2.4. Statistical Analysis

The collembolan abundance and diversity parameters (species richness, Shannon–Wiener index, and Pielou’s index) were calculated using Data Processing System (DPS) (version 2005 package, China).

As in our previous work [32], four collembolan morphological traits (ocelli number, body length, pigmentation level, and furcula development) were selected, as these traits reflect the feeding habits and moving patterns of Collembola [27]. Trait attribute values were assigned according to Santorufo et al. [25] (Table 1), and then the community-weighted mean trait attribute values (CWM) were calculated as follows:

CWM = ∑PiXi(1)

where Xi was the trait attribute of the i-th species and Pi was the relative abundance of the i-th species.

Two-way ANOVA analysis was performed using SPSS (version 23, IBM, Chicago, IL, USA) to evaluate the effects of maize variety and maize growth stage on collembolan abundance, diversity and collembolan morphological traits (CWM). The maize variety and maize growth stage were used as “between subject” factors. Collembolan abundance was log_10_(N + 1)-transformed before analyses to ensure normality and equal variances, and Levene’s test of equality for error variances and asymmetry were used to analyze the homogeneity of variance. Tukey’s HSD post hoc test (α = 0.05) was used when significant differences between treatments means occurred.

To better explore the impact of Bt maize variety on soil community composition, the relationship between environmental variables (including maize variety, maize growth stage, soil water content, root biomass, sampling year, and soil pH value) and the collembolan community composition was analyzed by redundancy analysis (RDA) using CANOCO (version 4.5, Microcomputer Power, New York, NY, USA) [43].

## 3. Results

### 3.1. Collembola Composition

A total of 3717 collembolan individuals, over 17 species, were collected in this study. The most dominant species in both maize varieties were the species *Thalassaphorura macrospinata* in both 2014 and 2015. *Isotomodes* sp.1 wasanormal species in both maize varieties in both years. The species *Desoria* sp.1 and *Entomobrya* sp.2 were normal species in both maize varieties in 2014 but were rare in 2015. Other species were categorized as relatively rare in both maize varieties in both years (Table 2).

### 3.2. Effects of Maize Variety and Maize Growth Stage on Collembolan Abundance and Diversity

**Figure 1:**
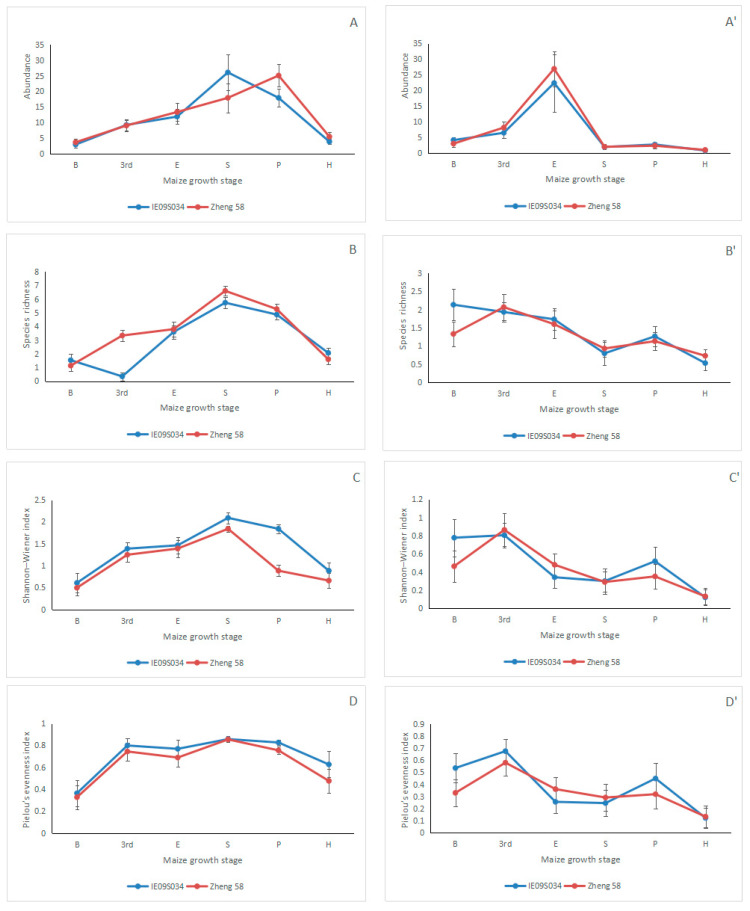
Collembolan abundances and diversity parameters (mean ± SE) in different maize variety fields (transgenic Bt maize IE09S034 and its near isoline of non-Bt maize Zheng 58) in Northeast China in 2014 and 2015. Maize growth stage “B”, “3^rd^”, “E”, “S”, “P”, and “H” are shorted for “before sowing”, “3rd leaf”, “elongation”, “silking”, “physiological maturity”, and “harvesting”, respectively. (**A**) is the collembolan abundance in 2014; (**A’**) is the collembolan abundance in 2015; (**B**) is the collembolan species richness in 2014; (**B’**) is the collembolan species richness in 2015; (**C**) is the collembolan Shannon-Wiener index in 2014; (**C’**) is the collembolan Shannon-Wiener index in 2015; (**D**) is the collembolan Pielou’s eveness index in 2014; (**D’**) is the collembolan Pielou’s eveness index in 2015.

ANOVA showed that maize variety had no significant effect on collembolan abundance (*N*), species richness (*S*), Shannon–Wiener index (*H’*), and Pielou’s evenness index (*J*) neither in 2014 nor in 2015 (Figure 1, Table 3), indicating that transgenic *cry1Ie* maize did not influence collembolan abundance and diversity. In addition, the results also showed that maize growth stage had significant effects on *N*, *S*, *H’*, and *J* of Collembola in the two varieties of maize plots, which indicated that the collembolan abundance and diversity were influenced by maize growth stage (Figure 1, Table 3).No effect of the interaction of maize variety × maize growth stage on *N*, *S*, *H’*, and *J* of Collembola was detected in neither year (Table 3).

### 3.3. Effects of Maize Variety and Maize Growth Stage on Collembolan Morphological TraitAttribute Value (CWM)

**Figure 2:**
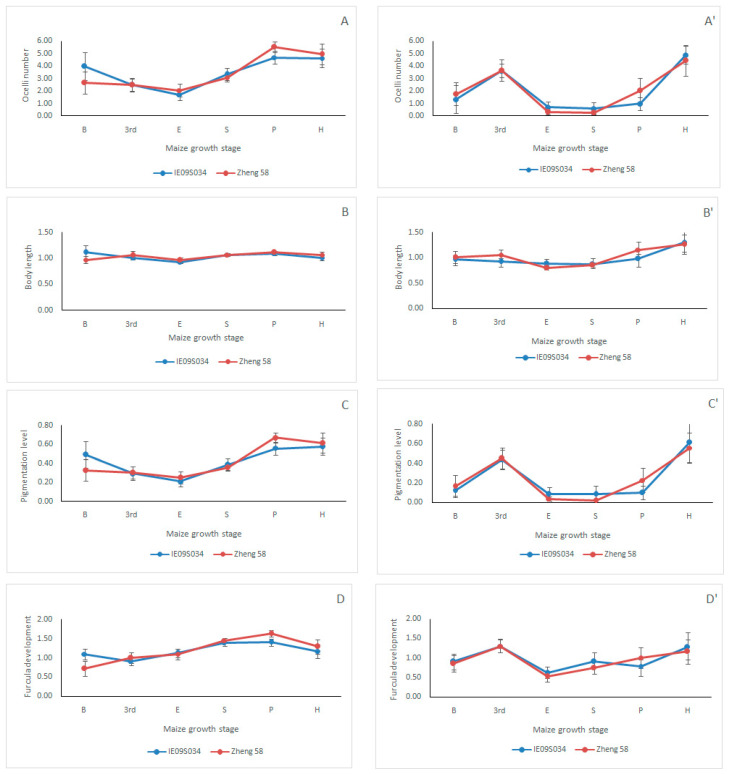
Collembolan morphological trait attribute values (CWM) (mean ± SE) in different maize variety fields (transgenic Bt maizeIE09S034 and its near-isoline of non-Bt maize Zheng 58) in Northeast China in 2014 and 2015. Maize growth stage “B”, “3rd”, “E”, “S”, “P”, and “H” are shorted for “before sowing”, “3rd leaf”, “elongation”, “silking”, “physiological maturity”, and “harvesting”, respectively. (**A**) is the collembolan ocelli number in 2014; (**A’**) is the collembolan ocelli number in 2015; (**B**) is the collembolan body length in 2014; (**B’**) is the collembolan body length in 2015; (**C**) is the collembolan pigmentation level in 2014; (**C’**) is the collembolan pigmentation level in 2015; (**D**) is the collembolan furcula development in 2014; (**D’**) is the collem-bolan furcula development in 2015.

ANOVA showed that maize variety had no significant effect on collembolan ocelli number (O), body length (B), pigmentation level (P), and furcula development (F) neither in 2014 nor in 2015 (Figure 2, Table 4), indicating that transgenic *cry1Ie* maize did not influence the evaluated collembolan morphological trait attribute values. In addition, the results also showed that maize growth stage had significant effects on O, B, P, and F of the Collembola in the two varieties of maize field, which indicated that the evaluated collembolan morphological trait attribute values were influenced by maize growth stage (Figure 2, Table 4). No effect of the interaction of maize variety × maize growth stage on O, B, P, and F of Collembola was detected in neither year (Table 4).

### 3.4. The Relationship between Environmental Variables and the Collembolan Community Composition

**Figure 3:**
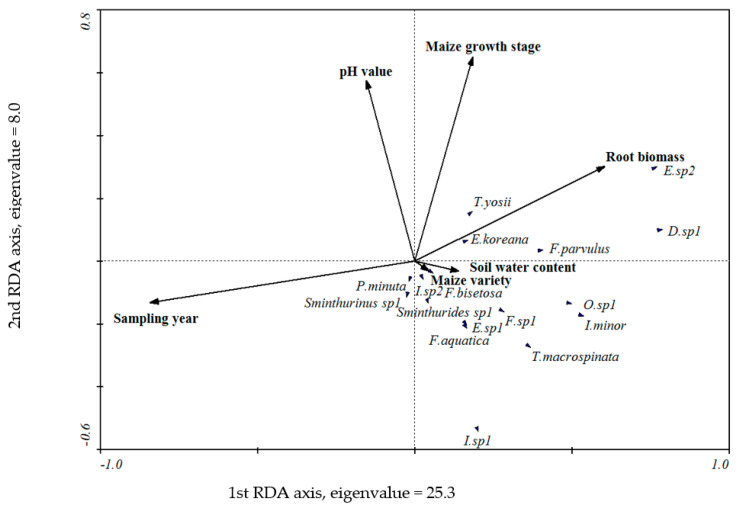
Redundancy analysis (RDA) for the relationship between environmental variables and the collembolan community composition.

Redundancy analysis showed that the environmental variables we selected (including sampling year, root biomass, maize growth stage, soil water content, pH value, and maize variety) explained 38% of the variation in the collembolan community composition. Among these variables, the sampling year (19%) explained the most, followed by the root biomass (7%), maize growth stage (6%), and pH value (4%). These variables affected the collembolan community composition significantly (Monte Carlo test, *p* = 0.002, 0.002, 0.002, and 0.010, respectively), while the soil water content (1%) and maize variety (1%) did not affect the collembolan community composition significantly (*p* = 0.166 and 0.666, respectively) (Figure 3, Table 5).

## 4. Discussion

It has been hypothesized that Bt crops may sometimes have a negative effect on soil ecosystems since they introduce Bt proteins or other secondary metabolites into the soil [44,45]. Collembola, as one of the three primary varieties of soil fauna, are sensitive to environmental changes [22,23], so they are often used as indicators of pollution and disturbance of soil ecosystems [46,47], and they have been also used in evaluating the effects of Bt crops on non-target soil fauna [48,49,50].

Collembolan abundance, species richness, Shannon–Wiener index, and Pielou’s evenness index are often used as measuring parameters in evaluating the effects of pollution and disturbance of soil ecosystems on collembolan community [36]. In our study, we also used these parameters, and the results clearly showed that maize variety had no significant effect on them neither in 2014 nor in 2015, indicating that transgenic *cry1Ie* maize did not influence the soil collembolan abundance and diversity. These results were consistent with most previous Bt corn studies [36,48,49,50]. For example, Bitzer et al. [48] reported that neither the estimated collembolan species richness nor Shannon–Wiener index differed significantly between Bt and its isoline maize using pitfall traps method in a 2-yr field trial; Al-Deeb et al. [51] found that Bt corn did not influence the collembolan abundance using tullgren-type extraction method in a 2-yr field study. The results of previous studies in China also indicated that Bt rice [49] and Bt cotton [50] did not influence the collembolan abundance and diversity.

However, the responses of different collembolan species to Bt maize cultivation may not be the same due to the differences of their ecological niches and feeding habitats, as laboratory experiments conducted by Bakonyi et al. [52] indicated that the effect of the Bt-toxin producing maize on the collembolan was species specific. So, sometimes, the abundance and diversity based on taxonomic approach may not reflect the collembolan community composition exactly. For example, although Arias-Martín et al. [12] found that cultivation of Bt maize does not negatively affect the total collembolan abundance and Shannon–Wiener index, some Collembola species were more abundant in Bt maize than in its isoline. Since different collembolan species distributed through different soil profiles have evolved differing morphological traits, usually the attribute values of collembolan ocelli number, body length, pigmentation level, and furcula development of euedaphic groups are at a lower level, those from semi-edaphic groups are at a medium level, and those from epedaphic groups are at a higher level [27]. Assessment of collembolan morphological traits could be a good supplement to abundance and diversity in assessing collembolan community responses to Bt maize cultivation [32]. Therefore, in the present study, we also analyzed some collembolan morphological trait attribute values. The results showed that maize variety had no significant effect on all the evaluated collembolan trait attribute values, indicating that the proportion of different Collembola groups occupying different niches was not influenced by maize variety; this supplemented the former results of collembolan abundance and diversity, and these two facts indicate that Bt maize IE09S034 did not influence collembolan community in Northeast China.

Many variables in the soil environment affect the soil fauna community structure, such as temperature, soil organic matter content, soil bulk density, pH value, and humidity [53,54,55]. In the present study, ANOVA showed that the effects of maize growth stage on collembolan abundance, diversity, and morphological trait values were all significant, indicating that maize growth stage significantly influenced the collembolan community, which may be caused by the different soil environment of different growing stage. The results of our RDA also showed that the sampling year, root biomass, maize growth stage, and pH value affect the collembolan community significantly; they explained the variables for the most part (36%). However, the maize variety explained the least of the variables (1%), it did not affect the collembolan community significantly, and this is important supporting evidence for the previous results of collembolan diversity and collembolan morphological traits.

## 5. Conclusions

In summary, maize variety did not influence collembolan abundance, diversity, and morphological trait values over the two consecutive years. RDA also indicated that maize variety did not affect collembolan community composition significantly. These results suggest that two years cultivation of *cry1Ie* maize does not affect the collembolan community in Northeast China. However, considering the limitation of time and area, longer time and bigger area field experiment is necessary in the future.

## Figures and Tables

**Table 1:** Attributes of collembolan traits.

Trait	Data Type	Attribute Value
Ocelli number	Ordinal	0–8
Body length	Quantitative	in mm, to the nearest 0.1mm
Pigmentation level	Ordinal	0 = neither colored nor patterned 1 = colored with no patterns 2 = colored with distinct patterns
Furcula development	Ordinal	0 = absent 1 = shorter than head 2 = longer than head

**Table 2:** Collembolan community composition in Bt maize IE09S034 and non-Bt maize Zong 31 in 2014 and 2015.

Collembola Group			2014				2015			
			Number		Percentage (%)		Number		Percentage (%)	
Family	Genus	Species	Bt	CK	Bt	CK	Bt	CK	Bt	CK
Onychiuridae	*Thalassaphorura*	*macrospinata*	278	252	25.69	17.91	265	296	45.93	45.47
Tullbergiidae	*Tullbergia*	*yosii*	4	7	0.37	0.50	1	1	0.17	0.15
Isotomidae	*Isotomodes*	sp.1	119	179	11.00	12.72	189	223	32.76	34.25
	*Isotomodes*	sp.2	2	0	0.18	0	0	0	0	0
	*Isotomiella*	*minor*	73	93	6.75	6.61	11	9	1.91	1.38
	*Folsomia*	*aquatica*	60	68	5.55	4.83	29	23	5.03	3.53
	*Folsomia*	*bisetosa*	7	23	0.65	1.63	4	1	0.69	0.15
	*Folsomides*	*parvulus*	29	24	2.68	1.71	9	0	1.56	0
	*Folsomides*	sp.1	4	6	0.37	0.43	1	1	0.17	0.15
	*Proisotoma*	*minuta*	13	22	1.20	1.56	6	7	1.04	1.08
	*Desoria*	sp.1	251	272	23.20	19.33	3	9	0.52	1.38
Entomobryidae	*Orchesellides*	sp.1	60	107	5.55	7.60	15	26	2.60	3.99
	*Entomobrya*	*koreana*	22	43	2.03	3.06	10	22	1.73	3.38
	*Entomobrya*	sp.1	13	38	1.20	2.70	28	26	4.85	3.99
	*Entomobrya*	sp.2	145	272	13.40	19.33	4	4	0.69	0.61
Sminthurididae	*Sminthurinus*	sp.1	2	0	0.18	0	0	2	0	0.31
	*Sminthurides*	sp.1	0	1	0	0.07	2	1	0.35	0.15
Total			1082	1407	100	100	577	651	100	100

**Table 3:** Effects of maize variety (Bt maize and non-Bt maize) and maize growth stage on soil collembolan abundance and diversity, analyzed using a two-way ANOVA analysis. The values highlighted in bold are statistically significant (*** *p* < 0.001).

Year.	Variable	Abundance (*N*)		Species Richness (*S*)		Shannon-Wiener index (*H’*)		Pielou’s Evenness index (*J*)	
		*F*	*P*	*F*	*P*	*F*	*P*	*F*	*P*
2014	Maize variety	2.422	0.122	0.020	0.887	0.482	0.489	1.872	0.173
	Maize growth stage	35.702	0.000 ***	41.94	0.000 ***	29.62	0.000 ***	10.925	0.000 ***
	Maize variety×Maize growth stage	0.437	0.822	0.903	0.480	0.445	0.816	0.203	0.961
2015	Maize variety	0.202	0.654	0.331	0.566	0.331	0.566	0.528	0.469
	Maize growth stage	15.075	0.000 ***	6.360	0.000 ***	6.039	0.000 ***	5.014	0.000 ***
	Maize variety×Maize growth stage	0.602	0.699	0.763	0.577	0.667	0.649	0.612	0.691

**Table 4:** Effects of maize variety (Bt maize and non-Bt maize) and maize growth stage on collembolan morphological trait attribute values (CWM), analyzed using a two-way ANOVA. The values highlighted in bold are statistically significant (* *p* < 0.05; ** *p* < 0.01; *** *p* < 0.001).

Year	Variable	Ocelli Number (O)		Body Length (B)		Pigmentation Level (P)		Furcula Development (F)	
		*F*	*P*	*F*	*P*	*F*	*P*	*F*	*P*
2014	Maize variety	0.002	0.964	0.005	0.946	0.000	0.993	0.043	0.836
	Maize growth stage	9.687	0.000 ***	2.408	0.039 *	9.388	0.000 ***	7.252	0.000 ***
	Maize variety×Maize growth stage	0.637	0.672	0.847	0.518	0.659	0.655	1.062	0.384
2015	Maize variety	0.018	0.893	0.252	0.617	0.002	0.963	0.076	0.783
	Maize growth stage	8.593	0.000 ***	3.088	0.012 *	8.741	0.000 ***	3.449	0.006 **
	Maize variety×Maize growth stage	0.272	0.928	0.399	0.848	0.269	0.929	0.192	0.965

**Table 5:** The effects of environmental variables on collembolan community by RDA (Monte Carlo test). The values highlighted in bold are statistically significant (* *p* < 0.05; ** *p* < 0.01).

Environmental Variable	Variance Explained (%)	*F*	*p*
Sampling year	19	16.11	0.002 **
Root biomass	7	6.55	0.002 **
Maize growth stage	6	6.56	0.002 **
Soil water content	4	3.40	0.010 *
pH value	1	1.49	0.166
Maize variety	1	0.66	0.666

## Data Availability

The data presented in this study are available on request from the corresponding author. The data are not publicly available due to privacy.
